# A Case of Cholecystotomy

**Published:** 1898-12

**Authors:** T. Campbell Grey


### A CASE OF CHOLECYSTOTOMY.

BY

T. Campbell Grey, F.R.C.S. Eng., L.R.C.P. Lond.

I have thought it worth while to record the following case, as the recovery from a serious condition of things was complete, and as the operation was done in a small cottage in the country.

Mrs. G., aged 43, who had suffered from symptoms of gallstones for four years, came under my care in April, 1898. She was then complaining of pain in the right side, over the region of the gall-bladder; she was slightly jaundiced, and under treatment improved somewhat, but speedily relapsed and the jaundice became very marked.

On one occasion, about the beginning of July, I was called to her about 1 a.m., and found her with typical symptoms of biliary colic.

From this time the jaundice increased, bile was entirely absent from the stools, the urine was very dark, she had more or less pain in her right side, and was losing flesh.

She was taking a saline purgative mixture every morning, and a wine-glassful of olive oil night and morning. She was obliged to discontinue the oil, owing to the nausea it produced.

I determined to do cholecystotomy, and to treat the stone in the duct as circumstances permitted.

Accordingly, on July 17th, 1898, Dr. Cross kindly administering chloroform, and with the assistance of Dr. W. H. Hillyer, I made a vertical incision downwards about 2½ inches long from the tip of the tenth rib on the right side; the peritoneum being reached and opened, I inserted my finger into the abdomen and felt a small contracted gall-bladder full of stones.

The gall-bladder was brought to the surface with a little difficulty, and being caught between two catch-forceps a small incision was made into it, through which a pair of dressing-forceps was passed, and four large stones were withdrawn with them. The gall-bladder contained some bile-stained mucus, and its walls were much thickened. Upon introducing my finger to explore the ducts a stone was felt in the common duct, which I endeavoured to push along towards the bowel: in this, however, I was not successful; my assistant, however, managed with a little manipulation to pass it on. The gall-bladder was united along the incision in it by a continuous suture to the abdominal wall, the abdominal cavity was then closed by two or three interrupted silkworm gut sutures above and below the attachment of the gall-bladder. The gall-bladder was drained with a rubber drainage-tube. The wound was then dressed with cyanide gauze and wool. There was no shock after the operation.

Her recovery was uneventful, she never had a bad symptom. The stools assumed their natural colour, and the jaundice disappeared slowly. She got up on the tenth day, and expressed herself as being freer from pain than for four years.

Only a drop or two of bile was discharged through the drainage-tube in the gall-bladder, which was left out on the fourteenth day. The wound was quite healed on the twenty-first day.

At the operation it was found that the gall-bladder was adherent to the omentum and bowel, and if the stone in the common duct had not yielded to persuasion I do not think, owing to the adhesions, that choledochotomy would have been possible.

The diagnosis in this case was difficult: as it was, one could exclude cancer of the gall-bladder alone, but whether there was cancer in conjunction with stones it was next to impossible to decide.

The chief point relied on to exclude cancer was that no enlargement of the gall-bladder could be felt.

